# Levels of Severity of Depressive Symptoms Among At-Risk Groups in the UK During the COVID-19 Pandemic

**DOI:** 10.1001/jamanetworkopen.2020.26064

**Published:** 2020-10-26

**Authors:** Eleonora Iob, Philipp Frank, Andrew Steptoe, Daisy Fancourt

**Affiliations:** 1 Research Department of Behavioural Science and Health, University College London, London, United Kingdom

## Abstract

**Importance:**

An immediate research priority is to investigate and monitor the psychological well-being among high-risk groups during the coronavirus disease 2019 (COVID-19) pandemic.

**Objective:**

To examine levels of severity of depressive symptoms over time among individuals with high risk in the UK during the COVID-19 pandemic.

**Design, Setting, and Participants:**

This cohort study is part of an ongoing large panel study of adults aged 18 years and older residing in the UK, the COVID-19 Social Study, established on March 21, 2020. Data analysis was conducted in May 2020.

**Exposures:**

Sociodemographic risk factors included belonging to the Black, Asian, and minority racial/ethnic communities, low socioeconomic position (SEP), and essential worker roles (eg, workers in health and social care, education, childcare, or key public services). Health-related and psychosocial risk factors included preexisting physical and mental health conditions, experience of psychological or physical abuse, and low social support.

**Main Outcomes and Measures:**

Depressive symptoms were measured on 7 occasions from March 21 to April 2, 2020, using the 9-item Patient Health Questionnaire (PHQ-9). Group-based depressive symptom trajectories were derived using latent growth mixture modeling.

**Results:**

The analytical sample comprised 51 417 adults aged 18 years and older (mean [SD] age, 48.8 [16.8] years; 26 276 [51.1%] women; 6145 members [12.0%] of Black, Asian, and minority racial/ethnic communities). Among these, 17 143 participants (33.3%) were in the lowest SEP quartile, and 11 342 participants (22.1%) were classified as essential workers. Three levels of severity of depressive symptoms were identified: low (30 850 participants [60.0%]), moderate (14 911 participants [29.0%]), and severe (5656 participants [11.0%]). After adjusting for covariates, experiences of physical or psychological abuse (odds ratio [OR], 13.16; 95% CI, 12.95-13.37; *P* < .001), preexisting mental health conditions (OR, 12.99; 95% CI, 12.87-13.11; *P* < .001), preexisting physical health conditions (OR, 3.41; 95% CI, 3.29-3.54; *P* < .001), low social support (OR, 12.72; 95% CI, 12.57-12.86; *P* < .001), and low SEP (OR, 5.22; 95% CI, 5.08-5.36; *P* < .001) were significantly associated with severe depressive symptoms. No significant association was found for race/ethnicity (OR, 1.07; 95% CI, 0.85-1.28; *P* = .56). Participants with essential worker roles were less likely to experience severe depressive symptoms (OR, 0.66; 95% CI, 0.53-0.80; *P* < .001). Similar patterns of associations were found for the group of participants with moderate depressive symptoms (abuse: OR, 5.34; 95% CI, 5.15-5.54; *P* < .001; mental health condition: OR, 4.24; 95% CI, 4.24-4.24; *P* < .001; physical health condition: OR, 1.89; 95% CI, 1.80-1.98; *P* < .001; low social support: OR, 4.71; 95% CI, 4.60-4.82; *P* < .001; low SEP: OR, 1.97; 95% CI, 1.87-2.08; *P* < .001).

**Conclusions and Relevance:**

In this cohort study of UK adults participating in the COVID-19 Social Study, people with psychosocial and health-related risk factors, as well as those with low SEP, were at the most risk of experiencing moderate or severe depressive symptoms during the COVID-19 pandemic.

## Key Points

**Question:**

Are sociodemographic, psychosocial, and health-related factors associated with risk of poor mental health during the COVID-19 pandemic in the UK?

**Findings:**

This cohort study using data from a large panel study including 51 417 adults found that the risks of moderate and severe depressive symptom trajectories were significantly higher among people experiencing abuse or low social support, individuals with low socioeconomic position, and those with preexisting mental and physical health conditions.

**Meaning:**

These findings suggest that mental health and socioeconomic interventions in the current or future pandemics should be targeted toward people with these risk factors.

## Teaser

This cohort study examines the levels of severity of depressive symptoms among at-risk individuals during the COVID-19 pandemic in the UK.

## Introduction

Owing to the rapid spread of the coronavirus disease 2019 (COVID-19), large numbers of people across the UK have been urged to stay at home (also known as *lockdown*) for potentially significant periods of time. It is already evident that the COVID-19 pandemic and its containment measures have profound implications for many aspects of society.^1^ Up to now, much focus has been placed on investigating the incidence and mortality rates of COVID-19, the pathogenesis of the virus, its adverse effects on physical health, and its increasing impact on the global economy. But there is increasing awareness of the implications of the pandemic for mental health.^2^ The consequences of the COVID-19 pandemic are likely to occur against a background of elevated prevalence of mental health problems in the UK. The widespread experience of COVID-19–related stressors (eg, loss of employment, illness or death of a relative), reduced access to mental health services, and frequent concerns about mental and physical health in the general population highlight the importance of identifying who is most at risk and how their experiences are evolving as the pandemic continues.

Previous studies on other epidemics and pandemics (eg, the severe acute respiratory syndrome epidemic in 2003 or the 2014 Ebola outbreak) suggest a plausible increase in mental health problems across the wider population, including symptoms of posttraumatic stress, confusion, anger and depression.^2,3^ However, it is unclear how mental health problems are manifesting in more at-risk groups during the current pandemic, such as in people with preexisting physical or mental health conditions, people with experiences of physical and psychological abuse, and people of lower socioeconomic position (SEP). Prior to the COVID-19 outbreak, the prevalence of depression was estimated at 4% to 5% in the general population,^4,5^ with considerably higher rates observed in individuals with distinct health-related, sociodemographic, and psychosocial risk factors.^6^ For example, the prevalence of depression in people with chronic obstructive pulmonary disease (COPD) has been placed at 27%^7^; type 2 diabetes, 18% to 20%^8,9^; myocardial infarction, 20%^10^; cancer, 13% to 17%^11,12^; and stroke, 29% to 33%.^13,14^ Furthermore, meta-analytic evidence indicates significantly elevated rates of depression in women who experienced intimate partner violence (27%),^15^ and almost a 2-fold increased risk of depression in people with lower SEP.^16^

Evidence already suggests that preexisting health inequalities may be strongly reflected in the current COVID-19 pandemic. For example, recent mortality statistics indicate that the severe acute respiratory syndrome coronavirus 2 (SARS-CoV-2), the virus that causes COVID-19, may be particularly detrimental to members of Black, Asian, and minority racial/ethnic groups, with approximately 19% of UK COVID-19–related hospital deaths occurring in members of these communities.^17^ In addition, essential workers (eg, frontline health care and social care staff) may be at particularly high risk of developing symptoms of emotional distress. According to a study of the psychosocial effects associated with severe acute respiratory syndrome in a sample of 510 hospital workers, 29% of health care staff reported elevated symptoms of emotional distress.^18^ The COVID-19 pandemic and related containment measures are also likely to accentuate social isolation and feelings of loneliness.^1^ Notably, these factors are themselves associated with the incidence, severity, and progression of negative mental health outcomes, including depression, anxiety-related disorders, and suicide.^19^ Other COVID-19–related stressors, such as loss of employment, financial hardship, overcrowded households, and diminished access to social support networks, may further contribute to disease burden, nationally and globally, by increasing levels of distress and reducing social opportunities relevant to both mental and physical well-being.^20^

Given the possibility that specific sociodemographic, psychosocial, and health-related characteristics may make some people at higher risk of worse mental health outcomes, an immediate research priority is to investigate the psychological well-being in these groups, and to understand how it evolves over time as the pandemic continues. Therefore, the aim of this study was to explore the severity levels of depressive symptoms among individuals at high risk in the UK during the COVID-19 pandemic.

## Methods

This cohort study is part of an ongoing large panel study of adults residing in the UK, the COVID-19 Social Study. Ethical approval for the COVID-19 Social Study was granted by the University College London ethics committee. All participants provided online informed consent. The study is General Data Protection Regulation compliant. This study is reported following the Strengthening the Reporting of Observational Studies in Epidemiology (STROBE) reporting guideline. The study protocol, along with more details on the sample, recruitment, response rate and weighting are published elsewhere.^21^

### Study Design and Participants

This study was established on March 21, 2020, using online weekly data collection to explore the psychological and social experiences of adults during the COVID-19 pandemic. The sample was recruited using 3 primary approaches. First, snowballing was used, including promoting the study through existing networks and mailing lists (including large databases of adults who had previously consented to be involved in health research across the UK), print and digital media coverage, and social media. Second, more targeted recruitment was undertaken focusing on individuals from a low-income background, individuals with no or few educational qualifications, and individuals who were unemployed. Third, the study was promoted via partnerships with third sector organizations to at-risk groups, including adults with preexisting mental health conditions, older adults, caretakers, and people experiencing domestic violence or abuse.

This study focused on participants recruited between March 21 and May 4, 2020. We ran a duplicate check through all email addresses in the database. Where duplicates were found, we retained the original sign-up from that email address and deleted the other accounts. All duplicate email addresses were removed prior to the sample size being calculated, and only individuals who provided at least 1 fully completed interview were included. This resulted in a final analytical sample of 51 417 participants. Although participant selection was based on a multistage nonrandom sampling approach, the sample was well-stratified across sociodemographic characteristics, and all data were weighted to the proportions of sex, age, race/ethnicity, education, and country of residence obtained from the Office for National Statistics.

### Measures

#### Depressive Symptoms

Depressive symptoms were measured using the 9-item Patient Health Questionnaire (PHQ-9), a validated screening tool for diagnosing depression in primary care.^22^ The questionnaire involves 9 questions about the frequency of experiencing common symptoms of major depressive disorder during the past week.^23^ Each item is answered on a 4-point Likert scale, ranging from not at all to nearly every day. Higher overall scores indicate more depressive symptoms, with scores of 0 to 4 suggesting minimal depression; 5 to 9, mild depression; 10 to 14, moderate depression; 15 to 19, moderately severe depression; and 20 to 27, severe depression.^24^

#### Exposures

##### Health-Related Risk Factors

Preexisting physical health conditions were measured by asking participants whether they had received a clinical diagnosis of diabetes, high blood pressure, heart disease, lung disease (eg, asthma or COPD), cancer, or another clinically diagnosed chronic physical health condition. Each item was answered on a binary response scale (yes = 1; no = 0). For the purposes of our analyses, a binary score was computed, distinguishing between the presence (≥1 preexisting physical health condition) and absence (no preexisting physical health condition) of a clinically diagnosed physical illness.

Preexisting mental health conditions were assessed by asking participants whether they had received a clinical diagnosis of depression, anxiety, or other mental health condition. Items were ranked on a binary response scale (yes = 1; no = 0). A summary score was computed, categorizing responses into the presence (≥1 clinically diagnosed mental health condition) vs absence (no preexisting mental health conditions) of a preexisting mental health condition.

##### Sociodemographic Risk Factors

Participants were asked to provide information about their racial/ethnic backgrounds. Race/ethnicity was categorized into 2 groups: White (ie, British, Irish, or other) vs Black, Asian, or minority racial/ethnic group (ie, Asian/Asian British, Black/Black British, White and Black/Black British, mixed race, Chinese/Chinese British, Middle Eastern/Middle Eastern British, other ethnic group).

A continuous latent SEP index was computed using 5 indicators of SEP: household income, employment status, education, household tenure, and household overcrowding. Income was measured by self-reported annual household income. Responses were categorized as less than £16 000 (<US $20 541) per year, £16 000 to £29 999 (US $20 541-$38 513) per year, £30 000 to £59 999 (US $38 514-$77 027) per year, £60 000 to 89 999 (US $77 028-$115 542) per year, and £90 000 or more (≥US $115 543) per year. Employment was measured by asking participants to provide information about their employment status. Responses were grouped as employed, inactive, and unemployed. Education was assessed in reference to participants’ highest educational qualification (ie, postgraduate, undergraduate, A-level or vocational, and General Certificate of Secondary Education or lower). Household tenure was categorized as owned outright, owned with mortgage or shared ownership, or rented. Overcrowded households were defined as households with less than 1 room per occupant. Next, we computed a binary SEP index variable by first dividing the continuous latent SEP variable into quartiles, and then categorizing the highest quartile as low SEP.

To identify individuals with essential worker roles, participants were asked whether they had currently fulfilled any of the government’s identified essential worker roles (yes = 1; no = 0). Essential workers were defined as people with jobs that were deemed essential during the pandemic and included those in health and social care, education and childcare, key public services, local and national government, public safety and national security, and transport, as well as utility workers. People were only classed as essential workers if their role involved them leaving the home to perform this work during the lockdown.

##### Psychosocial Risk Factors

Experience of psychological or physical abuse was determined based on participants response to whether during the last week they had been “physically harmed or hurt by someone else” or “bullied, controlled, intimidated, or psychologically hurt by someone else.” Responses were rated on a 4-point Likert scale ranging from not at all to nearly every day. A binary variable was created focusing on any response on either item that indicated any experience of abuse on at least 1 occasion (yes = 1; no = 0).

Social support was measured using an adapted version of the 6-item short form of Perceived Social Support Questionnaire.^25^ This includes 6 questions asking participants whether, during the past week, they had “experienced a lot of understanding and support from others,” “a very close person whose help [they] can always count on,” “people with whom [they] can spend time and do things together,” “if [they] get sick, [they] have friends and family who will take care of [them],” “if [they] feel down, [they] have people [they] can talk to without hesitation,” and whether they could “if necessary, [they] can easily borrow something [they] need from neighbours or friends.” Each item is rated on a 5-point scale from not true at all to very true, with higher scores indicating higher levels of perceived social support. Minor adaptations were made to the language in the scale to make it relevant to experiences during COVID-19 (eTable 1 in the Supplement). A sum score was computed by adding up the mean scores (across all available waves) of each social support question per individual and dividing it into quartiles. We subsequently derived a binary social support variable, defining low social support as scores in the lowest quartile.

### Statistical Analysis

Covariates included age, sex, and the presence or absence of a suspected COVID-19 diagnosis. Group-based trajectories of depressive symptoms were estimated using latent growth mixture (LGM) modeling.^26^ This data-driven modeling technique enables the identification of individuals with similar trajectories of depressive symptoms by computing classes (or trajectories) of mean values within homogenous subgroups over time. Next, multivariate logistic regression models were fitted to examine the associations of sociodemographic, psychosocial, and health-related risk factors with group-based depressive symptom trajectories. We first tested the individual associations of the risk factors by fitting a separate regression model for each exposure (model 1). Second, we fitted a model including all exposures simultaneously to assess their mutually adjusted associations (model 2). All estimates were adjusted for age, sex, and the presence or absence of a suspected COVID-19 diagnosis. The LGM models were fitted using robust maximum likelihood estimation to account for missing data and for the nonnormal distribution of the depression scores. The results of all multivariate logistic regression analyses are presented as adjusted odds ratios (ORs) with corresponding 95% CIs. Data management and descriptive analyses were conducted using R statistical software version 3.4.4. Multivariate logistic regression and LGM analyses were performed using Mplus statistical software version 7 (Muthén & Muthén). Further details about the LGM analysis and model fit are presented in the eAppendix and eTable 2 in the Supplement. *P* values were 2-sided, and statistical significance was set at *P* < .05. Data analysis was conducted in May 2020.

## Results

### Descriptive Statistics

The characteristics of the study participants at the first assessment are reported in the Table. The weighted and unweighted descriptive statistics at each wave are reported in eTable 3 and eTable 4 in the Supplement. The weighted sample included 51 417 participants (mean [SD] age, 48.8 [16.8] years; 26 276 [51.1%] women; 6145 members [12.0%] of Black, Asian, and minority racial/ethnic communities). There was a higher proportion of participants in the oldest age group (≥60 years, 16 464 participants [32.1%]) compared with the youngest groups (18-29 years, 9228 participants [17.9%]). A total of 30 888 participants (60.1%) were employed, and 11 342 participants (22.1%) were employed in essential worker roles. In contrast, 19 414 participants (37.8%) were inactive, and 1115 participants (2.2%) were unemployed. Most participants had General Certificate of Secondary Education or A-level qualifications (Table). There was a higher proportion of participants in the low- and medium-income groups compared with the highest ones. A total of 4196 participants (8.2%) lived in overcrowded households. A total of 19 655 participants (38.2%) had a preexisting physical condition, 10 219 participants (19.9%) reported having at least 1 mental health condition, and almost 1 in 3 participants (15 702 participants [30.5%]) had moderate or severe symptoms of depression at the first assessment (eFigure in the Supplement). A total of 5798 participants (11.3%) had experienced psychological or physical abuse.

**Table.** Weighted Characteristics of the Study Participants at First Assessment

Characteristic	No. (%) (N = 51 417)
Sex	
Women	26 276 (51.1)
Men	25 140 (48.9)
Age, y	
18-29	9228 (17.9)
30-44	11 972 (23.3)
45-59	13 723 (26.7)
≥60	16 494 (32.1)
Race/ethnicity	
Black, Asian, and minority racial/ethnic community^a^	6145 (12.0)
White	45 272 (88.0)
Employment status	
Employed	30 888 (60.1)
Inactive	19 414 (37.8)
Unemployed	1115 (2.2)
Education	
Postgraduate	6964 (13.5)
Undergraduate	10 453 (20.3)
A-level or vocational	17 362 (33.8)
GCSE or lower	16 638 (32.4)
Income, £	
Missing, No.	5308
<16 000	9704 (21.0)
16 000-30 000	12 846 (27.9)
30 000-60 000	14 434 (31.3)
60 000-90 000	5488 (11.9)
>90 000	3638 (7.9)
Overcrowding, yes	4196 (8.2)
Housing tenure	
Missing, No.	179
Own with mortgage	15 991 (31.2)
Own outright	16 016 (31.3)
Rent	19 231 (37.5)
Socioeconomic position index, quartile^b^	
First (highest)	8353 (16.2)
Second	9525 (18.5)
Third	16 395 (31.9)
Fourth (lowest)	17 143 (33.3)
Essential worker, yes	11 342 (22.1)
Physical health condition	19 655 (38.2)
Mental health condition	10 219 (19.9)
Low social support	13 235 (25.7)
Psychological or physical abuse	5798 (11.3)
COVID-19 symptoms	7618 (14.8)
Depressive symptoms^c^	
Minimal or mild	35 715 (69.5)
Moderate	12 451 (24.2)
Severe	3251 (6.3)
Psychiatric medications, yes	7726 (18.0)

Abbreviations: COVID-19, coronavirus disease 2019; GCSE, General Certificate of Secondary Education.

^a^ Includes Asian/Asian British, Black/Black British, White and Black/Black British, mixed race, Chinese/Chinese British, Middle Eastern/Middle Eastern British, and other racial/ethnic group.

^b^ Quartiles were computed prior to weighting so actual percentages vary from 25% in the weighted sample.

^c^ Measured using the Patient Health Questionnaire. Minimal or mild depression indicates a score of 0 to 9; moderate depression; 10 to 19; and severe depression, 20 or higher.

### Group-Based Depressive Symptom Trajectories

**Figure 1.**
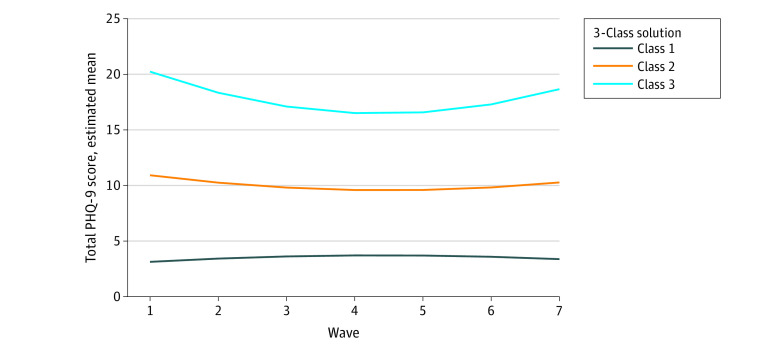
Group-Based Trajectories of Depressive Symptoms Class 1 indicates the low depressive symptom trajectory and includes participants with minimal depressive symptoms at all waves; class 2, the moderate depressive symptom trajectory and represents participants with moderate depressive symptoms throughout the study; class 3, the severe depressive symptom trajectory and represents participants with persistently severe levels of depressive symptoms. PHQ-9 indicates 9-item Patient Health Questionnaire.

The LGM analysis resulted in 3 distinct trajectories of depressive symptoms (Figure 1; eFigure and eTable 5 in the Supplement): class 1, indicating low depressive symptoms (including 30 850 participants [60.0%]); class 2, moderate depressive symptoms (including 14 911 participants [29.0%]); and class 3, severe depressive symptoms (including 5656 participants [11.0%]), which decreased following the start of lockdown but began to increase again in weeks 5 and 6.

### Associations of Sociodemographic, Health-Related, and Psychosocial Risk Factors With Depressive Symptom Trajectories

**Figure 2.**
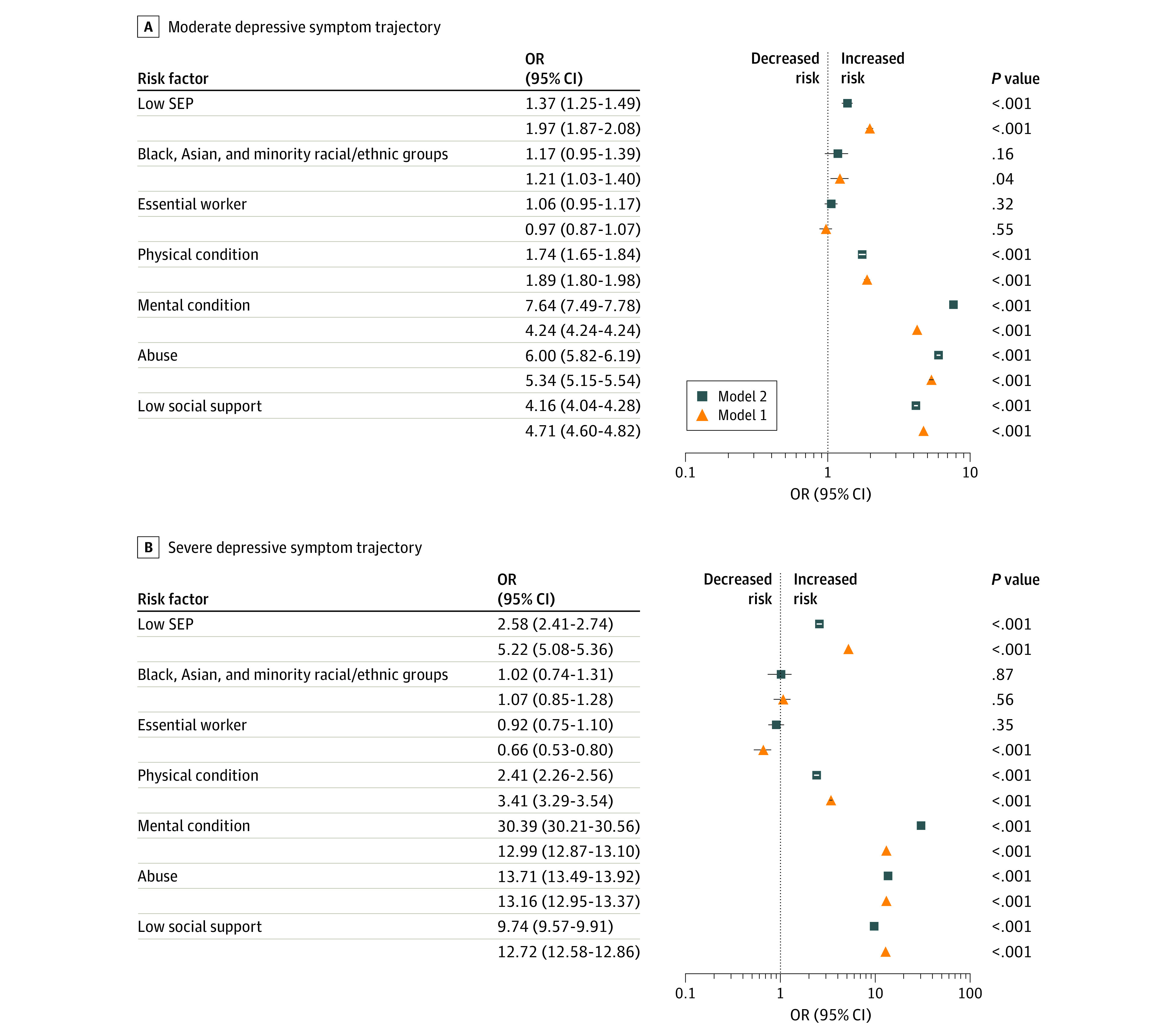
Associations of Sociodemographic, Psychosocial, and Health-Related Risk Factors With the Moderate and Severe Depressive Symptom Trajectories The odds ratios (ORs) represent the risk of belonging to the moderate or severe depressive symptom trajectory compared with the low trajectory and are plotted on the log scale. All models were adjusted for sex, age, and coronavirus disease 2019 diagnosis and weighted using survey weights. Model 1 further adjusted for each exposure, and model 2 adjusted for all exposures simultaneously. SEP indicates socioeconomic position.

The associations of the risk factors with levels of severity of depressive symptoms are presented in Figure 2 and eTable 6 in the Supplement. In model 1 adjusting for sex, age, and COVID-19 symptoms, low SEP was positively associated with moderate (OR, 1.97; 95% CI, 1.87-2.08; *P* < .001) and severe (OR, 5.22; 95% CI, 5.08-5.36; *P* < .001) depressive symptoms. These results maintained statistical significance when adjusting for other risk factors (model 2) (Figure 2). Belonging to the Black, Asian, or minority race/ethnicity community was associated with a greater risk of moderate depressive symptoms (OR, 1.21; 95% CI, 1.03-1.40; *P* = .04) but not severe depressive symptoms (OR, 1.07; 95% CI, 0.85-1.28; *P* = .56). However, these results were attenuated when adjusting for other risk factors. Participants with essential worker roles were less likely to experience severe depressive symptoms than those without such roles (OR, 0.66; 95% CI, 0.53-0.80; *P* < .001), but this result was not maintained when adjusting for other risk factors (Figure 2).

Health-related risk factors were associated with a higher likelihood of moderate and severe levels of depressive symptoms. The largest ORs were observed for preexisting mental health conditions (moderate depressive symptoms: OR, 4.24; 95% CI, 4.24-4.24; *P* < .001; severe depressive symptoms: OR, 12.99; 95% CI, 12.87-13.10; *P* < .001), followed by physical health conditions (moderate depressive symptoms: OR, 1.89; 95% CI, 1.79-1.98; *P* < .001; severe depressive symptoms: OR, 3.41; 95% CI, 3.29-3.54; *P* < .001). Psychosocial risk factors were also associated with an elevated risk of moderate and severe depressive symptoms. The ORs of moderate depressive symptoms were 5.34 (95% CI, 5.15-5.54) for participants who had experienced abuse (*P* < .001) and 4.71 (95% CI, 4.60-4.82) for participants with low social support (*P* < .001). In addition, the risk of severe depressive symptoms was more than 2-fold higher than that of moderate depressive symptoms among participants who experienced abuse (OR, 13.16; 95% CI, 12.95-13.37; *P* < .001) and those with low social support (OR, 12.72; 95% CI, 12.57-12.86; *P* < .001). These risk factors all remained statistically significant when adjusting for other risk factors (Figure 2).

### Sensitivity Analyses

We estimated the associations of the risk factors with the depressive symptom trajectories adjusting for the use of psychiatric medications (eTable 7 in the Supplement). Most estimates were broadly similar to those found in the models without adjustment for psychiatric medications, except for the association of preexisting mental health conditions with both moderate and severe depressive symptoms.

To assess the accuracy of the self-reported data on preexisting mental health conditions, we also compared PHQ-9 scores in participants with and without a preexisting mental health condition. This analysis showed that participants who reported a preexisting mental health condition had a mean PHQ-9 score more than 2-fold that of participants without preexisting mental health conditions at all time points (eTable 8 in the Supplement), providing some evidence for the validity and reliability of this measure.

## Discussion

To our knowledge, this cohort study is the first to investigate trajectories of depressive symptoms among high risk groups in the UK during the COVID-19 pandemic using data from a large longitudinal study. Our analysis revealed a number of key findings. First, trajectories of depressive symptoms in the sample were relatively constant across the first 6 weeks of lockdown. However, this does not imply that mental health has not been affected by the pandemic. Data from various surveys suggest that mental health worsened in the lead up to lockdown being introduced in the UK, leading to higher than usual levels of anxiety and depression.^27,28,29,30,31^ Although our analysis does not claim to show prevalence, owing to the nonrandom nature of the sample, it is notable that the mean scores presented in this study were substantially higher than PHQ-9 means previously reported in population estimates in other high income countries, such as the US.^32^ Furthermore, our results suggest that there was little improvement in depression in the first few weeks of lockdown.

Our findings also showed that individuals facing certain sociodemographic, psychosocial, and health-related risk factors were at heightened risk of experiencing moderate and severe depression during lockdown. The risks of moderate and severe depressive symptoms were higher among people experiencing abuse or low social support, in individuals with low SEP, and in those with preexisting mental or physical health conditions. Notably, these associations were different than might be expected from previous literature, particularly for psychosocial risk factors. For example, a meta-analytical results have suggested that the odds of depression are 1.87 (95% CI, 1.42-2.46) higher in people exposed to intimate partner violence compared with non-exposed people^15^ and 0.74 (95% CI, 0.72-0.76) lower in people with high levels of social support compared with those with poor social support.^33^ The associations of abuse and low social support with severe depressive symptoms were more than 5-fold larger in our study, with ORs of 13.2 for abuse and 12.7 for low social support. Likewise, previous meta-analyses have shown that low SEP increases the odds of depression by 1.81 (95% CI, 1.57-2.10) and chronic physical health conditions, such as cardiovascular disease, increase the odds of depression by 1.75 (95% CI, 1.36-2.26). Our data suggest that the odds of severe depressive symptoms were more than 3-fold higher for people with chronic physical conditions and more than 5-fold higher in those facing socioeconomic disadvantage. These figures are particularly worrying considering likely increases in rates of abuse, domestic violence, and unemployment, as well as diminished access to social networks during the COVID-19 pandemic.^3,34^ As such, the results presented here suggest that certain groups who usually experience higher odds of depression are at even greater risk during the current pandemic. In addition, it is plausible that both episodic and chronic psychological distress experienced during this pandemic may result in an increased incidence of various adverse physical health outcomes. For example, a 2016 longitudinal study from 17 countries^35^ reported that psychological distress was associated with subsequent diagnosis of stroke, heart disease, diabetes, chronic lung disease, chronic pain, and cancer. Hence, it is possible that depressive symptoms experienced during this pandemic may further increase disease risk, particularly in individuals with distinct sociodemographic, health-related, and psychosocial risk factors. Therefore, identifying individuals at high risk with elevated levels of depressive symptoms and providing evidence-based treatments to alleviate psychological distress may also be beneficial for preventing the development of physical illness.

Notably, belonging to the Black, Asian, or minority racial/ethnic community was associated with higher depressive symptoms, but these results were explained by other sociodemographic characteristics, abuse, and social support, as well as preexisting physical or mental conditions. Thus, it appears that the higher prevalence of other socioeconomic and psychosocial risk among racial/ethnic minority groups is a greater risk factor than race/ethnicity alone for depression during the pandemic. Additionally, despite evidence suggesting that people with essential worker roles, such as health care workers, are particularly susceptible to poor mental health outcomes during epidemics,^18^ our results show that the risk of elevated depressive symptoms was similar in participants with and without essential worker roles. However, our essential worker measure was not limited to health care professions but also included other essential worker roles, such as teachers and transport workers, who might be experiencing different levels of work-related stress during the COVID-19 pandemic. Furthermore, it is possible that self-selection bias determined these findings, with only essential workers who are psychologically coping with the current demands of their roles taking the time to participate in the research.

### Strengths and Limitations

This study has a number of strengths, including the large sample size, the longitudinal study design, and the statistical methods used to explore the associations between specific risk factors and depressive symptoms over time. However, our findings need to be interpreted in light of several limitations. First, our sample, although well-stratified across sociodemographic characteristics and weighted to population proportions, was not random, and hence is not nationally representative. It is possible that the study inadvertently attracted individuals experiencing greater psychological distress during the pandemic, or individuals who were more engaged or interested in mental health. Hence, the results shown here are not presented as prevalence figures but are instead used to understand risk factors. Second, the data used in this analysis are based on self-reported measures, bearing the risk of self-report bias. For example, a recent analysis of the agreement between self-reported health measures and administrative health records in the English Longitudinal Study of Aging found substantial underreporting of noncommunicable diseases in the self-reported data compared with the hospital record.^36^ Underreporting of mental health problems is also common in surveys owing to the social stigma associated with mental illness.^37^ Participants might have also underreported other sensitive information, such as experiences of abuse, especially if they were living with their abuser during lockdown. Hence, our results must be interpreted in light of these limitations. Nevertheless, our sensitivity analysis provides some evidence for the accuracy of the self-reported data on preexisting mental health conditions. Furthermore, causality cannot be assumed, as the study is observational and only provides information about the severity of depressive symptoms among high-risk groups over time. As we lack data on individuals from prior to lockdown being enacted in the UK, we are unable to identify whether and how patterns of depressive symptoms during lockdown vary relative to an individual’s usual mental health. However, we attempted to address this limitation by adjusting our model estimates for preexisting mental health conditions in the mutually adjusted analyses. In addition, we are unable to determine whether experiences of abuse increased during lockdown, as we lack data on earlier experiences of abuse. A previous history of abuse or trauma, as well as a genetic predisposition, are likely factors to be associated with an increased risk of poor mental health outcomes during the COVID-19 pandemic and should therefore be investigated in future studies.

## Conclusions

In this cohort study of UK adults participating in the COVID-19 Social Study, we found that certain at-risk groups are at increased risk of experiencing elevated depressive symptoms during the current COVID-19 pandemic, including people with preexisting mental and physical health conditions, experience of physical or psychological abuse, low social support, and low SEP. These groups may be experiencing even greater risk than in ordinary circumstances. In contrast, essential workers and individuals from racial/ethnic minority groups were not more likely to report depressive symptoms when other risk factors were taken into account. These differential associations highlight the importance of developing strategies to identify at-risk individuals, reallocate mental health services to those in need, and provide evidence-based treatments to alleviate depressive symptoms.

## Notes

Supplement. **eAppendix.** Supplemental Methods **eTable 1.** Comparison of Items in the Original and Revised Perceived Social Support Questionnaire **eTable 2.** LGM Model Fit Indices **eTable 3.** Weighted Characteristics of the Study Participants at Each Wave **eTable 4.** Unweighted Characteristics of the Study Participants at Each Wave **eTable 5.** Observed Depressive Symptom Scores at Each Wave by Latent Trajectory Group Membership **eTable 6.** Associations of Sociodemographic, Psychosocial, and Health-Related Risk Factors With Group-Based Trajectories of Depressive Symptoms **eTable 7.** Associations of Sociodemographic, Psychosocial, and Health-Related Risk Factors With Group-Based Trajectories of Depressive Symptoms, Adjusted for Psychiatric Medications **eTable 8.** Observed Depressive Symptom Scores (PHQ-9) at Each Wave by Preexisting Mental Illness **eFigure.** Observed PHQ-9 Mean Total Scores and SDs by Latent Trajectory Group Membership **eReference.**
