# Supplementary material for: Levels of Severity of Depressive Symptoms Among At-Risk Groups in the UK During the COVID-19 Pandemic
Source: JAMA Netw Open. 2020 Oct 26;3(10):e2026064. doi: 10.1001/jamanetworkopen.2020.26064 (PMC7588938; doi:10.1001/jamanetworkopen.2020.26064)
Supplement: Supplement. — eAppendix. Supplemental Methods eTable 1. Comparison of Items in the Original and Revised Perceived Social Support Questionnaire eTable 2. LGM Model Fit Indices eTable 3. Weighted Characteristics of the Study Participants at Each Wave eTable 4. Unweighted Characteristics of the Study Participants at Each Wave eTable 5. Observed Depressive Symptom Scores at Each Wave by Latent Trajectory Group Membership eTable 6. Associations of Sociodemographic, Psychosocial, and Health-Related Risk Factors With Group-Based Trajectories of Depressive Symptoms eTable 7. Associations of Sociodemographic, Psychosocial, and Health-Related Risk Factors With Group-Based Trajectories of Depressive Symptoms, Adjusted for Psychiatric Medications eTable 8. Observed Depressive Symptom Scores (PHQ-9) at Each Wave by Preexisting Mental Illness eFigure. Observed PHQ-9 Mean Total Scores and SDs by Latent Trajectory Group Membership eReference. [file jamanetwopen-e2026064-s001.pdf]

# Supplemental Online Content

Iob E, Frank P, Steptoe A, Fancourt D. Levels of severity of depressive symptoms among at-risk groups in the UK during the COVID-19 pandemic. *JAMA Netw Open*. 2020;3(10):e2026064. doi:10.1001/jamanetworkopen.2020.26064


**eAppendix.** Supplemental Methods
**eTable 1.** Comparison of Items in the Original and Revised Perceived Social Support Questionnaire
**eTable 2.** LGM Model Fit Indices
**eTable 3.** Weighted Characteristics of the Study Participants at Each Wave
**eTable 4.** Unweighted Characteristics of the Study Participants at Each Wave
**eTable 5.** Observed Depressive Symptom Scores at Each Wave by Latent Trajectory Group Membership
**eTable 6.** Associations of Sociodemographic, Psychosocial, and Health-Related Risk Factors With Group-Based Trajectories of Depressive Symptoms
**eTable 7.** Associations of Sociodemographic, Psychosocial, and Health-Related Risk Factors With Group-Based Trajectories of Depressive Symptoms, Adjusted for Psychiatric Medications
**eTable 8.** Observed Depressive Symptom Scores (PHQ-9) at Each Wave by Pre-existing Mental Illness
**eFigure.** Observed PHQ-9 Mean Total Scores and SDs by Latent Trajectory Group Membership
**eReference.**


This supplemental material has been provided by the authors to give readers additional information about their work.

© 2020 Iob E et al. *JAMA Network Open.*

## **eAppendix.** Supplemental Methods

### Latent Growth Mixture (LGM) Modelling

LGM models are characterised by the combination of latent growth curve modelling and latent class analysis. Such method allows to group individuals into trajectories according to their pattern of change over time. We estimated one LGM model with a specific intercept and slope growth factor for each trajectory. The within-group variation for each trajectory is represented by the intercept and slope growth factors which are modelled as continuous latent variables. The time metric was defined as *weeks into the study* (week 1- week 7). The optimal number of trajectories was identified using a stepwise approach. First, we fitted one LGM model with a single trajectory. Second, we included an additional trajectory at each following step until the optimal number of trajectories was reached. The optimal number of trajectories was determined using the following criteria: lower Akaike information criterion (AIC), lower sample-size adjusted Bayesian Information Criterion (ssaBIC), every class containing more than 5% of participants, entropy value (i.e. quality of the classification model) closest to 1 and > .80 (entropy values < 0.80 may indicate that the classes are not well separated, leading to convergence issues).[1] If the model with N +1 classes had one fit index that was worse than in the model with N classes, then the model with N classes was selected. In addition, we also evaluated the interpretability of the latent classes and whether they were theoretically meaningful for our research questions. The three-class solution was selected as the best fitting model since it had the best combination of lower AIC/ssaBIC and higher entropy, contained more than 5% of participants in each class, and had good interpretability (see sTable 2).

© 2020 Iob E et al. *JAMA Network Open.*

**eTable 1.** Comparison of Items in the Original and Revised Perceived Social Support Questionnaire

| **Original** | **Adapted for COVID-19**<br><br>**In the past week, I feel…** |
|---|---|
| I experience a lot of understanding and security from others | I have experienced a lot of understanding and support from others |
| I know a very close person whose help I can always count on | I have a very close person whose help I can always count on |
| If necessary, I can easily borrow something I might need from neighbours or friends | If necessary, I can easily borrow something I need from neighbours or friends |
| I know several people with whom I like to do things | I have people with whom I can spend time and do things together |
| When I am sick, I can without hesitation ask friends and family to take care of important matters for me | If I get sick, I have friends and family who will take care of me |
| If I am down, I know to whom I can go without hesitation | If I am feeling down, I have people I can talk to without hesitation |

© 2020 Iob E et al. *JAMA Network Open.*

**eTable 2.** LGM Model Fit Indices

| | **2 classes** | **3 classes** | **4 classes** | **5 classes** |
|---|---|---|---|---|
| *AIC* | 1015405.783 | **1009196.561** | 1005160.275 | 1002587.930 |
| *ssaBIC* | 1015542.660 | **1009379.064** | 1005388.404 | 1002861.685 |
| *N classes >5%* | Yes | **Yes** | Yes | No |
| *Entropy* | 0.813 | **0.811** | 0.768 | 0.771 |

© 2020 Iob E et al. *JAMA Network Open.*

**eTable 3.** Weighted Characteristics of the Study Participants at Each Wave

| | Wave 1 | Wave 2 | Wave 3 | Wave 4 | Wave 5 | Wave 6 | Wave 7 |
|---|---|---|---|---|---|---|---|
| | **Overall (N=51,417)** | **Overall (N=41,425)** | **Overall (N=34,314)** | **Overall (N=27,774)** | **Overall (N=18,009)** | **Overall (N=12,944)** | **Overall (N=406)** |
| **Sex** | | | | | | | |
| Female | 26276 (51.1%) | 21123 (51.0%) | 17463 (50.9%) | 14078 (50.7%) | 9278 (51.5%) | 6445 (49.8%) | 233 (57.4%) |
| Male | 25140 (48.9%) | 20302 (49.0%) | 16851 (49.1%) | 13696 (49.3%) | 8731 (48.5%) | 6499 (50.2%) | 173 (42.6%) |
| **Age** | | | | | | | |
| 18-29 | 9228 (17.9%) | 5918 (14.3%) | 3970 (11.6%) | 3024 (10.9%) | 1983 (11.0%) | 1186 (9.2%) | 92 (22.6%) |
| 30-44 | 11972 (23.3%) | 9287 (22.4%) | 7405 (21.6%) | 5664 (20.4%) | 3654 (20.3%) | 2439 (18.8%) | 130 (32.0%) |
| 45-59 | 13723 (26.7%) | 11565 (27.9%) | 9861 (28.7%) | 7921 (28.5%) | 5085 (28.2%) | 3636 (28.1%) | 111 (27.2%) |
| 60+ | 16494 (32.1%) | 14656 (35.4%) | 13078 (38.1%) | 11164 (40.2%) | 7287 (40.5%) | 5683 (43.9%) | 74 (18.1%) |
| **Ethnicity** | | | | | | | |
| BAME[a] | 6145 (12.0%) | 4176 (10.1%) | 3169 (9.2%) | 2490 (9.0%) | 1665 (9.2%) | 1080 (8.3%) | 60 (14.9%) |
| White | 45272 (88.0%) | 37249 (89.9%) | 31145 (90.8%) | 25283 (91.0%) | 16344 (90.8%) | 11864 (91.7%) | 346 (85.1%) |
| **Employment status** | | | | | | | |
| Employed | 30888 (60.1%) | 24386 (58.9%) | 19811 (57.7%) | 15614 (56.2%) | 10085 (56.0%) | 6925 (53.5%) | 284 (69.9%) |
| Inactive | 19414 (37.8%) | 16329 (39.4%) | 13940 (40.6%) | 11700 (42.1%) | 7640 (42.4%) | 5826 (45.0%) | 114 (28.0%) |
| Unemployed | 1115 (2.2%) | 710 (1.7%) | 564 (1.6%) | 460 (1.7%) | 284 (1.6%) | 193 (1.5%) | 8 (2.1%) |
| **Education** | | | | | | | |
| Postgraduate | 17362 (33.8%) | 13733 (33.2%) | 11153 (32.5%) | 8958 (32.3%) | 5768 (32.0%) | 4174 (32.2%) | 75 (18.5%) |
| Undergraduate | 16638 (32.4%) | 12958 (31.3%) | 10679 (31.1%) | 8620 (31.0%) | 5504 (30.6%) | 4151 (32.1%) | 59 (14.4%) |
| A-Level or Vocational | 6964 (13.5%) | 5984 (14.4%) | 5090 (14.8%) | 4169 (15.0%) | 2846 (15.8%) | 1896 (14.6%) | 159 (39.2%) |
| GCSE or Lower | 10453 (20.3%) | 8750 (21.1%) | 7392 (21.5%) | 6027 (21.7%) | 3890 (21.6%) | 2723 (21.0%) | 113 (27.9%) |
| **Income** | | | | | | | |
| N-Miss | 5308 | 4204 | 3513 | 2878 | 1891 | 1371 | 36 |

© 2020 Iob E et al. *JAMA Network Open.*

| | | | | | | | |
|---|---|---|---|---|---|---|---|
| <£16k | 9704 (21.0%) | 7510 (20.2%) | 6035 (19.6%) | 4857 (19.5%) | 3152 (19.6%) | 2380 (20.6%) | 28 (7.6%) |
| £16k - £30k | 12846 (27.9%) | 10270 (27.6%) | 8604 (27.9%) | 7005 (28.1%) | 4517 (28.0%) | 3372 (29.1%) | 76 (20.5 %) |
| £30k - £60k | 14434 (31.3%) | 11915 (32.0%) | 9956 (32.3%) | 8022 (32.2%) | 5131 (31.8%) | 3622 (31.3%) | 125 (33.8 %) |
| £60k - £90k | 5488 (11.9%) | 4547 (12.2%) | 3777 (12.3%) | 3030 (12.2%) | 1996 (12.4%) | 1328 (11.5%) | 74 (19.9 %) |
| >£90k | 3638 (7.9%) | 2979 (8.0%) | 2428 (7.9%) | 1981 (8.0%) | 1322 (8.2%) | 871 (7.5%) | 68 (18.3 %) |
| **Overcrowding** | | | | | | | |
| Not Overcrowded | 47221 (91.8%) | 38979 (94.1%) | 32574 (94.9%) | 26489 (95.4%) | 17201 (95.5%) | 12471 (96.3%) | 382 (93.9 %) |
| Overcrowded | 4196 (8.2%) | 2446 (5.9%) | 1740 (5.1%) | 1285 (4.6%) | 808 (4.5%) | 473 (3.7%) | 25 (6.1%) |
| **Tenure** | | | | | | | |
| N-Miss | 179 | 139 | 118 | 103 | 67 | 50 | 1 |
| Own Mortgage | 15991 (31.2%) | 13000 (31.5%) | 10617 (31.0%) | 8452 (30.5%) | 5375 (30.0%) | 3671 (28.5%) | 159 (39.1 %) |
| Own Outright | 16016 (31.3%) | 14011 (33.9%) | 12500 (36.6%) | 10546 (38.1%) | 6886 (38.4%) | 5311 (41.2%) | 101 (25.0 %) |
| Rent | 19231 (37.5%) | 14275 (34.6%) | 11078 (32.4%) | 8672 (31.3%) | 5681 (31.7%) | 3912 (30.3%) | 145 (35.8 %) |
| **Socioeconomic disadvantage index - quartiles** | | | | | | | |
| 4 (=highest) | 8353 (16.2%) | 6994 (16.9%) | 5867 (17.1%) | 4691 (16.9%) | 3079 (17.1%) | 2020 (15.6%) | 128 (31.6 %) |
| 3 | 9525 (18.5%) | 7689 (18.6%) | 6237 (18.2%) | 4934 (17.8%) | 3204 (17.8%) | 2193 (16.9%) | 119 (29.4 %) |
| 2 | 16395 (31.9%) | 13028 (31.4%) | 10685 (31.1%) | 8646 (31.1%) | 5546 (30.8%) | 3986 (30.8%) | 87 (21.4 %) |
| 1 (=lowest) | 17143 (33.3%) | 13714 (33.1%) | 11525 (33.6%) | 9502 (34.2%) | 6181 (34.3%) | 4746 (36.7%) | 71 (17.6 %) |
| **Essential worker** | | | | | | | |
| Yes | 11342 (22.1%) | 8737 (21.1%) | 6985 (20.4%) | 5407 (19.5%) | 3431 (19.1%) | 2375 (18.3%) | 75 (18.3 %) |
| **Chronic physical illness** | | | | | | | |
| Yes | 19655 (38.2%) | 16565 (40.0%) | 14094 (41.1%) | 11518 (41.5%) | 7444 (41.3%) | 5586 (43.2%) | 122 (29.9 %) |
| **Mental health disorder** | | | | | | | |
| Yes | 10219 (19.9%) | 7873 (19.0%) | 6160 (18.0%) | 4858 (17.5%) | 3084 (17.1%) | 2158 (16.7%) | 83 (20.5 %) |

© 2020 Iob E et al. *JAMA Network Open.*

| | | | | | | | |
|---|---|---|---|---|---|---|---|
| **Low social support** | | | | | | | |
| Yes | 13235 (25.7%) | 10496 (25.3%) | 8618 (25.1%) | 6920 (24.9%) | 4356 (24.2%) | 3230 (25.0%) | 103 (25.5 %) |
| **Psychological/physical abuse** | | | | | | | |
| Yes | 5798 (11.3%) | 4579 (11.1%) | 3802 (11.1%) | 3058 (11.0%) | 2008 (11.1%) | 1386 (10.7%) | 57 (14.0 %) |
| **COVID-19 symptoms** | | | | | | | |
| Yes | 7618 (14.8%) | 6237 (15.1%) | 5048 (14.7%) | 3984 (14.3%) | 2543 (14.1%) | 1794 (13.9%) | 78 (19.1 %) |
| **Depressive symptoms (categories)** | | | | | | | |
| N-Miss | 0 | 30 | 19 | 36 | 26 | 40 | 3 |
| minimal/mild | 35715 (69.5%) | 30078 (72.7%) | 26084 (76.1%) | 21330 (76.9%) | 14173 (78.8%) | 10019 (77.6%) | 284 (70.6 %) |
| moderate | 12451 (24.2%) | 9452 (22.8%) | 6845 (20.0%) | 5301 (19.1%) | 3152 (17.5%) | 2350 (18.2%) | 86 (21.4 %) |
| severe | 3251 (6.3%) | 1864 (4.5%) | 1366 (4.0%) | 1107 (4.0%) | 657 (3.7%) | 535 (4.1%) | 32 (8.0%) |

[a] BAME ethnic/racial background includes Asian/Asian British, Black/Black British, White and Black/Black British, Mixed race, Chinese/Chinese British, Middle Eastern/Middle Eastern British, other ethnic group. BAME = Black, Asian and minority ethnic groups.

© 2020 Iob E et al. *JAMA Network Open.*

**eTable 4.** Unweighted Characteristics of the Study Participants at Each Wave

| | Wave 1 | Wave 2 | Wave 3 | Wave 4 | Wave 5 | Wave 6 | Wave 7 |
|---|---|---|---|---|---|---|---|
| | **Overall (N=53,170)** | **Overall (N=45,028)** | **Overall (N=37,820)** | **Overall (N=30,273)** | **Overall (N=19,548)** | **Overall (N=13,668)** | **Overall (N=627)** |
| **Sex** | | | | | | | |
| Female | 39652 (74.6%) | 33478 (74.3%) | 28122 (74.4%) | 22568 (74.5%) | 14774 (75.6%) | 10202 (74.6%) | 505 (80.5%) |
| Male | 13518 (25.4%) | 11550 (25.7%) | 9698 (25.6%) | 7705 (25.5%) | 4774 (24.4%) | 3466 (25.4%) | 122 (19.5%) |
| **Age** | | | | | | | |
| 18-29 | 5126 (9.6%) | 3757 (8.3%) | 2718 (7.2%) | 2095 (6.9%) | 1392 (7.1%) | 836 (6.1%) | 64 (10.2%) |
| 30-44 | 14927 (28.1%) | 12287 (27.3%) | 10045 (26.6%) | 7763 (25.6%) | 5075 (26.0%) | 3319 (24.3%) | 222 (35.4%) |
| 45-59 | 17497 (32.9%) | 14999 (33.3%) | 12848 (34.0%) | 10330 (34.1%) | 6674 (34.1%) | 4653 (34.0%) | 222 (35.4%) |
| 60+ | 15620 (29.4%) | 13985 (31.1%) | 12209 (32.3%) | 10085 (33.3%) | 6407 (32.8%) | 4860 (35.6%) | 119 (19.0%) |
| **Ethnicity** | | | | | | | |
| BAME [a] | 2690 (5.1%) | 2036 (4.5%) | 1604 (4.2%) | 1263 (4.2%) | 845 (4.3%) | 538 (3.9%) | 35 (5.6%) |
| White | 50480 (94.9%) | 42992 (95.5%) | 36216 (95.8%) | 29010 (95.8%) | 18703 (95.7%) | 13130 (96.1%) | 592 (94.4%) |
| **Employment status** | | | | | | | |
| Employed | 35207 (66.2%) | 29458 (65.4%) | 24561 (64.9%) | 19471 (64.3%) | 12682 (64.9%) | 8541 (62.5%) | 479 (76.4%) |
| Inactive | 17226 (32.4%) | 15007 (33.3%) | 12795 (33.8%) | 10439 (34.5%) | 6634 (33.9%) | 4970 (36.4%) | 144 (23.0%) |
| Unemployed | 737 (1.4%) | 563 (1.3%) | 464 (1.2%) | 363 (1.2%) | 232 (1.2%) | 157 (1.1%) | 4 (0.6%) |
| **Education** | | | | | | | |
| Postgraduate | 9641 (18.1%) | 7992 (17.7%) | 6568 (17.4%) | 5215 (17.2%) | 3303 (16.9%) | 2377 (17.4%) | 37 (5.9%) |
| Undergraduate | 7706 (14.5%) | 6186 (13.7%) | 5086 (13.4%) | 4014 (13.3%) | 2506 (12.8%) | 1859 (13.6%) | 23 (3.7%) |
| A-Level or Vocational | 14336 (27.0%) | 12447 (27.6%) | 10647 (28.2%) | 8654 (28.6%) | 5856 (30.0%) | 3931 (28.8%) | 327 (52.2%) |
| GCSE or Lower | 21487 (40.4%) | 18403 (40.9%) | 15519 (41.0%) | 12390 (40.9%) | 7883 (40.3%) | 5501 (40.2%) | 240 (38.3%) |
| **Income** | | | | | | | |
| N-Miss | 4812 | 4071 | 3457 | 2792 | 1816 | 1305 | 36 |
| <£16k | 7279 (15.1%) | 5943 (14.5%) | 4895 (14.2%) | 3897 (14.2%) | 2433 (13.7%) | 1808 (14.6%) | 38 (6.4%) |

© 2020 Iob E et al. *JAMA Network Open.*

| | | | | | | | |
|---|---|---|---|---|---|---|---|
| £16k - £30k | 11570 (23.9%) | 9752 (23.8%) | 8139 (23.7%) | 6493 (23.6%) | 4176 (23.6%) | 3007 (24.3%) | 100 (16.9%) |
| £30k - £60k | 16718 (34.6%) | 14290 (34.9%) | 12024 (35.0%) | 9615 (35.0%) | 6185 (34.9%) | 4273 (34.6%) | 216 (36.5%) |
| £60k - £90k | 7530 (15.6%) | 6463 (15.8%) | 5466 (15.9%) | 4352 (15.8%) | 2855 (16.1%) | 1917 (15.5%) | 128 (21.7%) |
| >£90k | 5261 (10.9%) | 4509 (11.0%) | 3839 (11.2%) | 3124 (11.4%) | 2083 (11.7%) | 1358 (11.0%) | 109 (18.4%) |
| **Overcrowding** | | | | | | | |
| Not Overcrowded | 50457 (94.9%) | 43121 (95.8%) | 36375 (96.2%) | 29175 (96.4%) | 18842 (96.4%) | 13260 (97.0%) | 612 (97.6%) |
| Overcrowded | 2713 (5.1%) | 1907 (4.2%) | 1445 (3.8%) | 1098 (3.6%) | 706 (3.6%) | 408 (3.0%) | 15 (2.4%) |
| **Tenure** | | | | | | | |
| N-Miss | 184 | 143 | 123 | 106 | 68 | 48 | 2 |
| Own Mortgage | 19826 (37.4%) | 16782 (37.4%) | 14056 (37.3%) | 11131 (36.9%) | 7213 (37.0%) | 4867 (35.7%) | 277 (44.3%) |
| Own Outright | 18017 (34.0%) | 15963 (35.6%) | 13918 (36.9%) | 11403 (37.8%) | 7291 (37.4%) | 5412 (39.7%) | 188 (30.1%) |
| Rent | 15143 (28.6%) | 12140 (27.0%) | 9723 (25.8%) | 7633 (25.3%) | 4976 (25.5%) | 3341 (24.5%) | 160 (25.6%) |
| **Socioeconomic disadvantage index - quartiles** | | | | | | | |
| 4 (=highest) | 15151 (28.5%) | 12962 (28.8%) | 10978 (29.0%) | 8705 (28.8%) | 5756 (29.4%) | 3789 (27.7%) | 272 (43.4%) |
| 3 | 11283 (21.2%) | 9529 (21.2%) | 7989 (21.1%) | 6432 (21.2%) | 4189 (21.4%) | 2838 (20.8%) | 160 (25.5%) |
| 2 | 13701 (25.8%) | 11566 (25.7%) | 9607 (25.4%) | 7713 (25.5%) | 4929 (25.2%) | 3509 (25.7%) | 119 (19.0%) |
| 1 (=lowest) | 13035 (24.5%) | 10971 (24.4%) | 9246 (24.4%) | 7423 (24.5%) | 4674 (23.9%) | 3532 (25.8%) | 76 (12.1%) |
| **Essential worker** | | | | | | | |
| Yes | 12817 (24.1%) | 10502 (23.3%) | 8602 (22.7%) | 6660 (22.0%) | 4305 (22.0%) | 2875 (21.0%) | 160 (25.5%) |
| **Chronic physical illness** | | | | | | | |
| Yes | 19358 (36.4%) | 16715 (37.1%) | 14175 (37.5%) | 11420 (37.7%) | 7310 (37.4%) | 5304 (38.8%) | 183 (29.2%) |
| **Mental health disorder** | | | | | | | |

© 2020 Iob E et al. *JAMA Network Open.*

| | | | | | | | |
|---|---|---|---|---|---|---|---|
| Yes | 10174 (19.1%) | 8320 (18.5%) | 6759 (17.9%) | 5256 (17.4%) | 3317 (17.0%) | 2289 (16.7%) | 122 (19.5%) |
| **Low social support** | | | | | | | |
| Yes | 11593 (21.8%) | 9740 (21.6%) | 8174 (21.6%) | 6520 (21.5%) | 4112 (21.0%) | 2984 (21.8%) | 143 (22.8%) |
| **Psychological/physical abuse** | | | | | | | |
| Yes | 5422 (10.2%) | 4689 (10.4%) | 4052 (10.7%) | 3281 (10.8%) | 2134 (10.9%) | 1424 (10.4%) | 69 (11.0%) |
| **COVID-19 symptoms** | | | | | | | |
| Yes | 8295 (15.6%) | 7084 (15.7%) | 5917 (15.6%) | 4710 (15.6%) | 3023 (15.5%) | 2062 (15.1%) | 122 (19.5%) |
| **Depressive symptoms (categories)** | | | | | | | |
| N-Miss | 0 | 31 | 32 | 43 | 26 | 43 | 3 |
| minimal/mild | 38588 (72.6%) | 33642 (74.8%) | 29179 (77.2%) | 23505 (77.8%) | 15503 (79.4%) | 10681 (78.4%) | 455 (72.9%) |
| moderate | 12109 (22.8%) | 9770 (21.7%) | 7407 (19.6%) | 5761 (19.1%) | 3441 (17.6%) | 2486 (18.2%) | 139 (22.3%) |
| severe | 2473 (4.7%) | 1585 (3.5%) | 1202 (3.2%) | 964 (3.2%) | 578 (3.0%) | 458 (3.4%) | 30 (4.8%) |

[a] BAME ethnic/racial background includes Asian/Asian British, Black/Black British, White and Black/Black British, Mixed race, Chinese/Chinese British, Middle Eastern/Middle Eastern British, other ethnic group. BAME = Black, Asian and minority ethnic groups.

© 2020 Iob E et al. *JAMA Network Open.*

**eTable 5.** Observed Depressive Symptom Scores at Each Wave by Latent Trajectory Group Membership

| | Low Depression Trajectory | | Moderate Depression Trajectory | | High Depression Trajectory | |
|---|---|---|---|---|---|---|
| | Mean | SD | Mean | SD | Mean | SD |
| Wave 1 | 2.534 | 2.257 | 8.730 | 3.503 | 17.865 | 4.368 |
| Wave 2 | 2.531 | 2.121 | 8.650 | 3.182 | 17.285 | 4.207 |
| Wave 3 | 2.265 | 2.044 | 8.340 | 3.160 | 17.080 | 4.320 |
| Wave 4 | 2.139 | 1.995 | 8.291 | 3.281 | 17.264 | 4.409 |
| Wave 5 | 2.001 | 2.012 | 8.052 | 3.343 | 17.216 | 4.447 |
| Wave 6 | 2.172 | 2.218 | 8.456 | 3.608 | 17.472 | 4.564 |
| Wave 7 | 2.788 | 2.605 | 8.983 | 3.895 | 17.254 | 4.547 |

**Note.** The Low Depressive symptom trajectory (Class 1) includes participants with minimal depressive symptoms at all waves [30,850 individuals (60%)]. The Moderate depressive symptom trajectory (Class 2) represents participants with moderate depressive symptoms throughout the study [14,911 individuals (29%)]; The Severe depressive symptom trajectory (Class 3) represents participants with persistently severe levels of depressive symptoms [5,656 individuals (11%)].

© 2020 Iob E et al. *JAMA Network Open.*

**eTable 6.** Associations of Sociodemographic, Psychosocial, and Health-Related Risk Factors With Group-Based Trajectories of Depressive Symptoms

| | Moderate Depression Trajectory | | | | | | | | High Depression Trajectory | | | | | | | |
|---|---|---|---|---|---|---|---|---|---|---|---|---|---|---|---|---|
| | **OR** | **SE** | **p-value** | **95% CI - lower bound** | **95% CI - upper bound** | **Log OR** | **Log OR 95% CI - lower bound** | **Log OR 95% CI - upper bound** | **OR** | **SE** | **p-value** | **95% CI - lower bound** | **95% CI - upper bound** | **Log OR** | **Log OR 95% CI - lower bound** | **Log OR 95% CI - upper bound** |
| *Model 1 (individual effects)* | | | | | | | | | | | | | | | | |
| **Covariates** | | | | | | | | | | | | | | | | |
| Sex:Female | 1.74 | 0.044 | 0.000 | 1.66 | 1.83 | 0.24 | 0.16 | 0.33 | 1.64 | 0.061 | 0.000 | 1.52 | 1.76 | 0.216 | 0.096 | 0.096 |
| Age:30-44 | 0.57 | 0.071 | 0.000 | 0.43 | 0.71 | -0.24 | -0.38 | -0.11 | 0.40 | 0.079 | 0.000 | 0.25 | 0.56 | -0.397 | -0.552 | -0.552 |
| Age:45-59 | 0.38 | 0.072 | 0.000 | 0.24 | 0.52 | -0.42 | -0.56 | -0.28 | 0.29 | 0.078 | 0.000 | 0.14 | 0.44 | -0.539 | -0.692 | -0.692 |
| Age:60+ | 0.17 | 0.080 | 0.000 | 0.02 | 0.33 | -0.76 | -0.92 | -0.61 | 0.09 | 0.098 | 0.000 | -0.10 | 0.28 | -1.055 | -1.247 | -1.247 |
| ref: 18-29 | | | | | | | | | | | | | | | | |
| COVID-19 symptoms | 1.90 | 0.055 | 0.000 | 1.79 | 2.01 | 0.28 | 0.17 | 0.39 | 2.30 | 0.072 | 0.000 | 2.16 | 2.45 | 0.363 | 0.222 | 0.222 |
| **Socioeconomic vulnerabilities** | | | | | | | | | | | | | | | | |
| Low SEP | 1.97 | 0.054 | 0.000 | 1.87 | 2.08 | 0.30 | 0.19 | 0.40 | 5.22 | 0.072 | 0.000 | 5.08 | 5.36 | 0.718 | 0.577 | 0.577 |
| Ethnicity: BAME[a] | 1.21 | 0.093 | 0.038 | 1.03 | 1.40 | 0.19 | 0.01 | 0.38 | 1.07 | 0.109 | 0.562 | 0.85 | 1.28 | 0.027 | -0.186 | 0.915 |
| Essential worker | 0.97 | 0.050 | 0.545 | 0.87 | 1.07 | -0.01 | -0.11 | 0.08 | 0.66 | 0.070 | 0.000 | 0.53 | 0.80 | -0.179 | -0.316 | -0.316 |
| **Health-related vulnerabilities** | | | | | | | | | | | | | | | | |
| Physical illness | 1.89 | 0.046 | 0.000 | 1.79 | 1.98 | 0.28 | 0.19 | 0.37 | 3.41 | 0.064 | 0.000 | 3.29 | 3.54 | 0.533 | 0.407 | 0.407 |
| Mental illness | 4.24 | 0.000 | 0.000 | 4.24 | 4.24 | 0.63 | 0.63 | 0.63 | 12.99 | 0.058 | 0.000 | 12.87 | 13.10 | 1.114 | 1.000 | 1.000 |
| **Psychosocial vulnerabilities** | | | | | | | 0.00 | 0.00 | | | | | | | | |
| Abuse | 5.34 | 0.100 | 0.000 | 5.15 | 5.54 | 0.73 | 0.53 | 0.92 | 13.16 | 0.108 | 0.000 | 12.95 | 13.37 | 1.119 | 0.907 | 0.907 |
| Low social support | 4.71 | 0.056 | 0.000 | 4.60 | 4.82 | 0.67 | 0.56 | 0.78 | 12.72 | 0.073 | 0.000 | 12.57 | 12.86 | 1.104 | 0.961 | 0.961 |
| **Model 2 (mutually adjusted effects)** | | | | | | | | | | | | | | | | |
| **Covariates** | | | | | | | | | | | | | | | | |
| Sex:Female | 2.11 | 0.051 | 0.000 | 2.01 | 2.21 | 0.33 | 0.23 | 0.43 | 2.05 | 0.080 | 0.000 | 1.90 | 2.21 | 0.313 | 0.156 | 0.156 |
| Age:30-44 | 0.43 | 0.081 | 0.000 | 0.28 | 0.59 | -0.36 | -0.52 | -0.20 | 0.23 | 0.108 | 0.000 | 0.02 | 0.44 | -0.643 | -0.854 | -0.854 |
| Age:45-59 | 0.22 | 0.082 | 0.000 | 0.06 | 0.38 | -0.66 | -0.82 | -0.50 | 0.10 | 0.112 | 0.000 | -0.12 | 0.32 | -1.013 | -1.233 | -1.233 |
| Age:60+ | 0.09 | 0.097 | 0.000 | -0.10 | 0.28 | -1.06 | -1.25 | -0.87 | 0.02 | 0.143 | 0.000 | -0.26 | 0.30 | -1.689 | -1.970 | -1.970 |
| (ref: 18-29) | | | | | | | | | | | | | | | | |
| COVID-19 symptoms | 1.92 | 0.064 | 0.000 | 1.80 | 2.05 | 0.28 | 0.16 | 0.41 | 2.25 | 0.090 | 0.000 | 2.08 | 2.43 | 0.353 | 0.177 | 0.177 |
| **Socioeconomic vulnerabilities** | | | | | | | | | | | | | | | | |
| Low SEP | 1.37 | 0.061 | 0.000 | 1.25 | 1.49 | 0.14 | 0.02 | 0.26 | 2.58 | 0.084 | 0.000 | 2.41 | 2.74 | 0.411 | 0.247 | 0.247 |
| Ethnicity: BAME[a] | 1.17 | 0.111 | 0.161 | 0.95 | 1.39 | 0.07 | -0.15 | 0.29 | 1.02 | 0.147 | 0.870 | 0.74 | 1.31 | 0.010 | -0.278 | 1.428 |
| Essential worker | 1.06 | 0.056 | 0.315 | 0.95 | 1.17 | 0.02 | -0.09 | 0.13 | 0.92 | 0.089 | 0.351 | 0.75 | 1.09 | -0.036 | -0.210 | 0.477 |
| **Health-related vulnerabilities** | | | | | | | | | | | | | | | | |
| Physical illness | 1.74 | 0.050 | 0.000 | 1.65 | 1.84 | 0.24 | 0.14 | 0.34 | 2.41 | 0.076 | 0.000 | 2.26 | 2.56 | 0.383 | 0.234 | 0.234 |
| Mental illness | 7.64 | 0.073 | 0.000 | 7.49 | 7.78 | 0.88 | 0.74 | 1.03 | 30.39 | 0.090 | 0.000 | 30.21 | 30.56 | 1.483 | 1.306 | 1.306 |
| **Psychosocial vulnerabilities** | | | | | | | | | | | | | | | | |

© 2020 Iob E et al. *JAMA Network Open.*

| | | | | | | | | | | | | | | | |
|---|---|---|---|---|---|---|---|---|---|---|---|---|---|---|---|
| Abuse | 6.00 | 0.095 | 0.000 | 5.82 | 6.19 | 0.78 | 0.59 | 0.96 | 13.71 | 0.110 | 0.000 | 13.49 | 13.92 | 1.137 | 0.921 | 0.921 |
| Low social support | 4.16 | 0.060 | 0.000 | 4.04 | 4.28 | 0.62 | 0.50 | 0.74 | 9.74 | 0.087 | 0.000 | 9.57 | 9.91 | 0.988 | 0.818 | 0.818 |

© 2020 Iob E et al. *JAMA Network Open.*

**eTable 7.** Associations of Sociodemographic, Psychosocial, and Health-Related Risk Factors With Group-Based Trajectories of Depressive Symptoms, Adjusted for Psychiatric Medications

| | **Moderate Depression Trajectory** | | | | | | | | **High Depression Trajectory** | | | | | | | |
|---|---|---|---|---|---|---|---|---|---|---|---|---|---|---|---|---|
| | **OR** | **SE** | **p-value** | **95% CI - lower bound** | **95% CI - upper bound** | **Log OR** | **Log OR 95% CI - lower bound** | **Log OR 95% CI - upper bound** | **OR** | **SE** | **p-value** | **95% CI - lower bound** | **95% CI - upper bound** | **Log OR** | **Log OR 95% CI - lower bound** | **Log OR 95% CI - upper bound** |
| **Model 2 (mutually adjusted effects)** | | | | | | | | | | | | | | | | |
| **Covariates** | | | | | | | | | | | | | | | | |
| Sex:Female | 2.10 | 0.054 | 0.000 | 2.00 | 2.21 | 0.74 | 0.64 | 0.85 | 2.04 | 0.090 | 0.000 | 1.86 | 2.22 | 0.71 | 0.54 | 0.89 |
| Age:30-44 | 0.50 | 0.091 | 0.000 | 0.32 | 0.68 | -0.70 | -0.88 | -0.52 | 0.25 | 0.124 | 0.000 | 0.00 | 0.49 | -1.40 | -1.64 | -1.15 |
| Age:45-59 | 0.23 | 0.092 | 0.000 | 0.05 | 0.41 | -1.46 | -1.64 | -1.28 | 0.09 | 0.127 | 0.000 | -0.16 | 0.34 | -2.39 | -2.64 | -2.14 |
| Age:60+ | 0.09 | 0.108 | 0.000 | -0.13 | 0.30 | -2.45 | -2.66 | -2.24 | 0.02 | 0.160 | 0.000 | -0.29 | 0.33 | -3.98 | -4.30 | -3.67 |
| (ref: 18-29) | 1.90 | 0.070 | 0.000 | 1.76 | 2.04 | 0.64 | 0.51 | 0.78 | 2.52 | 0.098 | 0.000 | 2.33 | 2.71 | 0.92 | 0.73 | 1.12 |
| COVID-19 symptoms | | | | | | | | | | | | | | | | |
| **Socioeconomic vulnerabilities** | | | | | | | | | | | | | | | | |
| Low SEP | 1.37 | 0.061 | 0.000 | 1.25 | 1.49 | 0.14 | 0.02 | 0.26 | 2.58 | 0.084 | 0.000 | 2.41 | 2.74 | 0.41 | 0.25 | 0.25 |
| Ethnicity: BAME [a] | 1.18 | 0.125 | 0.188 | 0.93 | 1.42 | 0.16 | -0.08 | 0.41 | 1.07 | 0.173 | 0.716 | 0.73 | 1.40 | 0.06 | -0.28 | 0.40 |
| Essential worker | 1.03 | 0.060 | 0.670 | 0.91 | 1.14 | 0.03 | -0.09 | 0.14 | 0.91 | 0.100 | 0.325 | 0.71 | 1.10 | -0.10 | -0.30 | 0.10 |
| **Health-related vulnerabilities** | | | | | | | | | | | | | | | | |
| Physical illness | 1.67 | 0.054 | 0.000 | 1.56 | 1.77 | 0.51 | 0.40 | 0.62 | 2.13 | 0.083 | 0.000 | 1.97 | 2.29 | 0.76 | 0.59 | 0.92 |
| Mental illness | 3.95 | 0.091 | 0.000 | 3.77 | 4.13 | 1.37 | 1.19 | 1.55 | 13.50 | 0.115 | 0.000 | 13.28 | 13.73 | 2.60 | 2.38 | 2.83 |
| **Psychosocial vulnerabilities** | | | | | | | | | | | | | | | | |
| Abuse | 5.28 | 0.091 | 0.000 | 5.10 | 5.46 | 1.66 | 1.49 | 1.84 | 11.47 | 0.113 | 0.000 | 11.25 | 11.69 | 2.44 | 2.22 | 2.66 |
| Low social support | 4.25 | 0.063 | 0.000 | 4.12 | 4.37 | 1.45 | 1.32 | 1.57 | 9.62 | 0.096 | 0.000 | 9.43 | 9.81 | 2.26 | 2.08 | 2.45 |
| **Psychiatric medications** | 3.08 | 0.089 | 0.000 | 2.91 | 3.25 | 1.13 | 0.95 | 1.30 | 4.01 | 0.113 | 0.000 | 3.79 | 4.24 | 1.39 | 1.17 | 1.61 |

**Note.** The odds ratios represent the risk of belonging to the Moderate or Severe depressive symptom trajectory compared with the Low trajectory. All models were adjusted for sex, age, COVID-19 symptoms, and psychiatric medications and weighted using survey weights. [a] BAME ethnic/racial background includes Asian/Asian British, Black/Black British, White and Black/Black British, Mixed race, Chinese/Chinese British, Middle Eastern/Middle Eastern British, other ethnic group. BAME = Black, Asian and minority ethnic groups.

© 2020 Iob E et al. *JAMA Network Open.*

**eTable 8.** Observed Depressive Symptom Scores (PHQ-9) at Each Wave by Pre-existing Mental Illness

| | Mental illness = No | | Mental illness = Yes | |
|---|---|---|---|---|
| | Mean | Standard Deviation | Mean | Standard Deviation |
| Wave 1 | 5.601 | 5.022 | 12.124 | 6.732 |
| Wave 2 | 5.424 | 4.769 | 11.416 | 6.414 |
| Wave 3 | 5.056 | 4.679 | 11.018 | 6.441 |
| Wave 4 | 4.910 | 4.716 | 10.959 | 6.564 |
| Wave 5 | 4.654 | 4.681 | 10.553 | 6.559 |
| Wave 6 | 4.795 | 4.801 | 10.847 | 6.612 |
| Wave 7 | 5.706 | 5.064 | 11.008 | 6.508 |

© 2020 Iob E et al. *JAMA Network Open.*

**eFigure.** Observed PHQ-9 Mean Total Scores and SDs by Latent Trajectory Group Membership

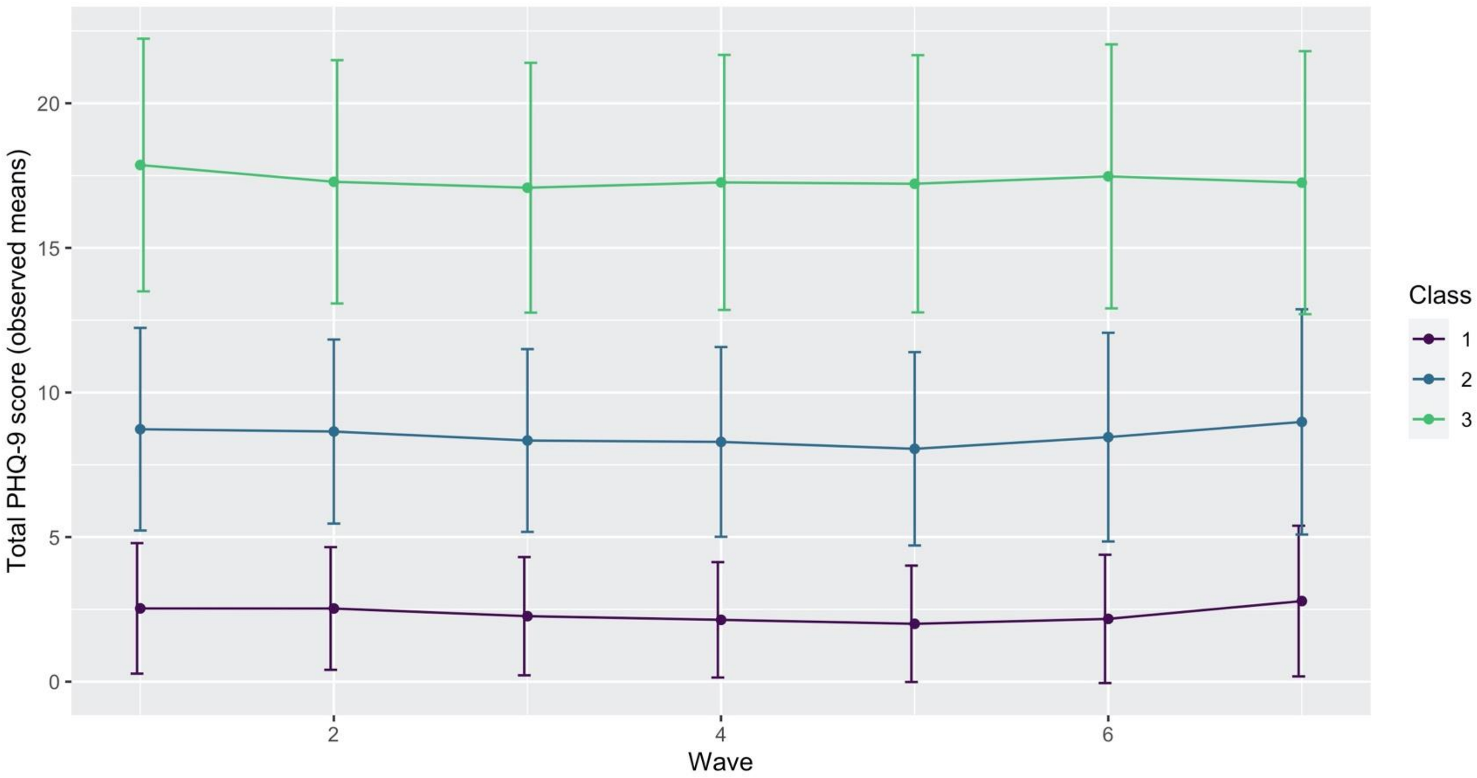


The Low Depressive symptom trajectory (Class 1) includes participants with minimal depressive symptoms at all waves [30,850 individuals (60%)]. The Moderate depressive symptom trajectory (Class 2) represents participants with moderate depressive symptoms throughout the study [14,911 individuals (29%)]; The Severe depressive symptom trajectory (Class 3) represents participants with persistently severe levels of depressive symptoms [5,656 individuals (11%)].

© 2020 Iob E et al. *JAMA Network Open.*

## eReference.

1. Herle M, Micali N, Abdulkadir M, et al. Identifying typical trajectories in longitudinal data: modelling strategies and interpretations. *European journal of epidemiology*. 2020:1-18.

© 2020 Iob E et al. *JAMA Network Open.*
